# International Paediatric Mitochondrial Disease Scale

**DOI:** 10.1007/s10545-016-9948-7

**Published:** 2016-06-09

**Authors:** Saskia Koene, Jan C. M. Hendriks, Ilse Dirks, Lonneke de Boer, Maaike C. de Vries, Mirian C. H. Janssen, Izelle Smuts, Cheuk-Wing Fung, Virginia C. N. Wong, I. René F. M. de Coo, Katharina Vill, Claudia Stendel, Thomas Klopstock, Marni J. Falk, Elizabeth M. McCormick, Robert McFarland, Imelda J. M. de Groot, Jan A. M. Smeitink

**Affiliations:** 1 Radboudn Center for Mitochondrial Medicine at the Department of Paediatrics, Radboudumc, Geert Grooteplein 10. 6500 HB, PO BOX 9101, Nijmegen, The Netherlands; 2 Department of Health Evidence, Radboudumc, Nijmegen, The Netherlands; 3 Steve Biko Academic Hospital, Ludwig-Maximilians-of Pretoria, Pretoria, South Africa; 4 Department of Paediatrics & Adolescent Medicine, University of Hong Kong, Hong Kong, Hong Kong; 5 Department of Neurology, ErasmusMC, Rotterdam, The Netherlands; 6 Department of Pediatric Neurology and Developmental Medicine, Dr. v. Hauner Children’s Hospital, Ludwig-Maximilians-University, Munich, Germany; 7 Department of Neurology, Friedrich-Baur-Institute, Ludwig-Maximilians-University, Munich, Germany; 8 German Center for Neurodegenerative Diseases (DZNE), Munich, Germany; 9 Munich Cluster for Systems Neurology (SyNergy), Munich, Germany; 10 Division of Human Genetics, Department of Pediatrics, The Children’s Hospital of Philadelphia, Philadelphia, PA 19104 USA; 11 Department of Pediatrics, University of Pennsylvania Perelman School of Medicine, Philadelphia, PA 19104 USA; 12 Wellcome Trust Centre for Mitochondrial Research Newcastle, Newcastle upon Tyne, UK; 13 Department of Rehabilitation, Radboudumc, Nijmegen, The Netherlands

## Abstract

**Objective:**

There is an urgent need for reliable and universally applicable outcome measures for children with mitochondrial diseases. In this study, we aimed to adapt the currently available Newcastle Paediatric Mitochondrial Disease Scale (NPMDS) to the International Paediatric Mitochondrial Disease Scale (IPMDS) during a Delphi-based process with input from international collaborators, patients and caretakers, as well as a pilot reliability study in eight patients. Subsequently, we aimed to test the feasibility, construct validity and reliability of the IPMDS in a multicentre study.

**Methods:**

A clinically, biochemically and genetically heterogeneous group of 17 patients (age 1.6–16 years) from five different expert centres from four different continents were evaluated in this study.

**Results:**

The feasibility of the IPMDS was good, as indicated by a low number of missing items (4 %) and the positive evaluation of patients, parents and users. Principal component analysis of our small sample identified three factors, which explained 57.9 % of the variance. Good construct validity was found using hypothesis testing. The overall interrater reliability was good [median intraclass correlation coefficient for agreement between raters (ICC_agreement_) 0.85; range 0.23–0.99).

**Conclusion:**

In conclusion, we suggest using the IPMDS for assessing natural history in children with mitochondrial diseases. These data should be used to further explore construct validity of the IPMDS and to set age limits. In parallel, responsiveness and the minimal clinically important difference should be studied to facilitate sample size calculations in future clinical trials.

**Electronic supplementary material:**

The online version of this article (doi:10.1007/s10545-016-9948-7) contains supplementary material, which is available to authorized users.

## Introduction

Mitochondrial diseases (MD) are the most prevalent inherited metabolic diseases, with an incidence of ∼1:5000 live births (Schaefer et al. 2004). Since mitochondria are present in almost all cells, theoretically, symptoms can arise from every organ. The most commonly affected organs and tissues include the brain, eye, heart, and skeletal muscle (Koopman et al. 2012). There is enormous variability in the pattern of affected organs and degree of disability experienced. Whereas some children with MD thrive in mainstream school and live well into adult life, others follow a more rapidly progressive course and die in the neonatal period or function at a low level, barely interacting with their environment.

Currently, there is no cure for MD, but there are some promising results of pharmacological interventions in cells and animals, and the prospects for randomised clinical trials of novel and repurposed pharmaceuticals are increasing (Koene and Smeitink 2009; Wenz 2009; Viscomi et al. 2011, 2015; Koopman et al. 2012; Blanchet et al. 2015; Peng et al. 2015). Outcome measures that are valid, reliable, sensitive and clinically relevant are critical to the success of such trials, but the heterogeneity and multisystemic nature of MD pose significant challenges in choosing an appropriate, universally applicable outcome measure (Koene et al. 2013a). To be able to measure disease severity and progression within the full range of the phenotypic spectrum, a combination of objective, subjective, functional and biochemical end points will probably be necessary.

An MD-specific follow-up tool for use with children already exists: the Newcastle Paediatric Mitochondrial Disease Scale (NPMDS) (Phoenix et al. 2006). This scale was originally designed to be a concise and pragmatic clinical tool to monitor the biophysical markers of disease progression. Although the NPMDS fits this purpose from a natural disease course perspective, it was not designed as an end-point instrument and probably lacks a sufficient level of detail required for this purpose in clinical trials. Moreover, the scale was not developed to measure the clinically relevant concept of functional disability (Phoenix et al. 2006).

In this study, we aimed to adapt the NPMDS to a more clinically relevant and detailed scoring system for future clinical trials in paediatric patients with MD, thus creating the International Paediatric Mitochondrial Disease Scale (IPMDS), which should cover more symptoms indicated by patients and parents as “burdensome,” such as tiredness and lack of energy, behavioural problems and depression (Koene et al. 2013b). We also aimed to include a functional domain to quantify changes in a child’s motor abilities, since clinically relevant changes in motor function are not always equally reflected by changes in muscle power or tone and vice versa (Abel et al. 2003; Beenakker et al. 2005; Parreira et al. 2010). After a Delphi-based development process, we tested the construct validity and reliability (interrater, intrarater and test–retest) by field testing in several expert international centres.

## Methods

The IPMDS was developed during a Delphi-based process by consulting patients, parents and MD experts. After the pilot reliability test, the scale was further optimised for subsequent testing in five expert centres. All raters received both written and video instructions. At each centre, two to four randomly selected patients were assessed by three to four physicians subsequently to evaluate interrater reliability. Construct validity was tested using factor analysis and by hypothesis testing. In a subset of patients within these studies, test–retest reliability and intrarater reliability was tested. For a more detailed description of the methods, we refer to supplementary file 1.

This study was conducted in all indicated countries after approval from the regional Medical Research Ethics Committee (MREC NL.44833.091.13). In accordance with the Declaration of Helsinki, written informed consent was obtained from each participant or his/her legal guardian(s).

### Statistics

Because of the relatively small number of patients in our study, we used nonparametric tests for analyses and report medians and ranges. Parent and patient experiences were assessed for each patient individually. From a rater perspective, the number of blank items was counted to test feasibility. Factor analysis was used to explain the variance–covariance matrix for data in terms of relationships between a much smaller number of unobserved variables, called factors. We used data from the rater with the least missing data, and missing items were replaced using the mean of the other three raters. We removed items for which <80 % was completed, including items that could be assessed only when the child was >6 years old (hopping, running, and rotating) and in case the child was unable to report complaints, such as headache, gastroesophageal reflux, muscle pain, vibration or subtle touch at physical examination. Items with little variance (less than two items score 1 or more) were also removed for the factor analysis. Principal component analysis (PCA) was used as the extraction method for factors. The orthogonal rotation (varimax) with Kaiser’s normalisation was used to simplify the interpretation of factors. The number of factors extracted was based on Eigen values >1. Sampling adequacy was determined using the Kaiser-Meyer-Olkin (KMO) measure. In addition, Bartlett’s test of sphericity was applied to test whether correlations between items were sufficiently large for PCA. The percentage of variance explained by each factor is also presented. Sum scores for the factors were calculated using clinically suitable items. Cronbach’s alpha was calculated as a measure of internal consistency of each constructed factor. The difference between patients with mild and with severe disease (median value rated by physicians) for these sum scores was tested using the Mann–Whitney* U* test.

We used hypothesis testing to assess construct validity. Since all mentioned hypotheses involved two measurements of the same construct, we aimed at moderate to good correlations (*ρ* = 0.4–0.79). We used Spearman’s correlation coefficients to correlate between continuous or interval variables (IPMDS and its subdomains and functional parameters). The mean raters’ score in each patient was used to calculate the correlation. Interrater reliability between physicians within one centre seeing one patient was calculated using intraclass correlation coefficient for agreement between raters (ICC_agreement_). Intrarater reliability within two physicians was calculated using ICC_agreement_ between each rater’s scores. Test–retest reliability was calculated using ICC_agreement_ between scores. An ICC_agreement_ ≥ 0.7 was used as “acceptable” (Vet et al. 2011).

We used a *p* value of 0.05 for statistical significance. Correlation coefficients were interpreted in accordance with the guidelines provided at the BMJ website (http://www.bmj.com/about-bmj/resources-readers/publications/statistics-square-one/11-correlation-and-regression). All analyses were performed using IBM’s SPSS Statistics 22.0.0.1.

## Results

### Cohort description

A clinically and genetically heterogeneous cohort of 17 children aged 1.6–16 years from five expert centres participated in this study (supplementary Table 4A). Rater details are presented in supplementary document 4B.

### Scale description

After our Delphi-based process, the IPMDS consists of 61 items in three domains: 23 in Domain 1 (subjective complaints and symptoms; obtained by interviewing parents); 25 in Domain 2 (physical examination; obtained by physical examination) and 13 in Domain 3 (functional assessment; obtained by physical/motor function evaluation). The final scale and the manual are presented in supplementary documents 2 and 3.

### Feasibility

The average time to complete the IPMDS was 35 min; 96 % of all items (85–100 %) relevant to the patient based on age and/or mental capacities was completed. Sixteen (patients and their) parents filled out the feasibility questionnaire. All patients and parents indicated that the number of questions, the burden of the physical examination to the child, the duration of the interview, physical examination and the total time burden were just right or could be (much) longer. The parents of a 2-year-old patient experienced difficulties in translating the questions to the situation of a toddler. This was not reflected in the number of missing items for toddlers (94 %; 91–97 %) and in the interrater reliability for the first and the third domain (ICC_agreement_ 0.86 versus 0.74 and 0.96 versus 0.97, respectively, for toddlers versus all children). The interrater reliability of the second domain was low in toddlers compared with the whole cohort (ICC_agreement_ 0.39 versus 0.81).

### Factor analysis

A total of 44 of 61 items was included in the factor analysis. Based on factor loadings and communalities, we were able to include 32 of the 49 items in one of the factors (Table 1). Items with little variance were removed (1.17 diarrhoea, 2.03 alertness, 2.04 breathing, 2.09 nystagmus). Sample adequacy of the factor analysis was sufficient for all items within the IPMDS (KMO 0.51). Bartlett’s tests of sphericity indicated that correlation between items was sufficiently large for PCA (*p* <0.001).We identified three factors (Tables 1 and 2), naming them based on items they contained: 1, basic functioning (31.1 % of explained variance); 2, eating and digesting (14.1 % of the explained variance); 3, abnormalities at neurological examination (12.7 % of explained variance). The internal consistency of these factors was good for factor 1 (Cronbach’s alpha = 0.97), acceptable for factor 2 (Cronbach’s alpha = 0.76) and poor for factor 3 (Cronbach’s alpha = 0.16). The total percentage of explained variance by the three factors was 57.9 %. Spearman’s correlation coefficients between factors (Table 3) indicate that all factors represent a unique identity.

**Table 1:** Factor analysis: factor loadings for individual items. Based on clinical communalities between items, we composed three factors (in bold) including 32 of the 49 items

Factor	Basic functioning	Eating and digesting	Abnormalities at neurological examination
3.05 Sitting	**0.926**		
3.11 Reaching	**0.916**		
3.12 Grasping	**0.901**		
1.13 Communicating	**0.871**		
3.07 Standing	**0.875**		
3.04 Sitting up	**0.867**		
2.18 Hypertonia	**0.848**		
3.08 Walking	**0.847**		
3.06 Standing up	**0.843**		
3.02 Head control	**0.831**		
3.03 Rolling over	**0.821**		
2.20 Rigidity	**0.808**		
3.01 Communication	**0.772**		0.513
1.22a Continence day	**0.592**		
1.18 Cognitive	**0.547**		0.486
2.11 Prox muscle	**0.408**	0.614	
1.11 Swallowing		**0.894**	
1.10 Chewing	0.536	**0.764**	
1.14 Vomiting		**0.74**	0.476
2.01 Growth		**0.507**	−0.405
1.16 Constipation		−**0.49**	
2.02 Weight	−0.404	**0.448**	
2.08 Eye movements			−**0.698**
2.16 Tremor			−**0.628**
2.13 Hypokinesia	0.745		**0.568**
1.01 Response			**0.543**
2.19 Hypotonia			**0.525**
2.15 Ataxia			−**0.511**
1.23a Strabismus			−**0.498**
2.14 Abnormal movement	0.69		−**0.496**
1.12 Hearing	0.403		**0.494**
2.17 Reflexes			**0.419**
1.22b Continence night		0.75	
2.07 Strabismus		0.741	
2.10 Vision		0.717	
1.02 Exercise			0.616
1.09 Infections			
1.04 Tired			
1.23b Ptosis			
1.06 Epilepsy			
1.21 Breathing			
2.06 Ptosis			

Only factor loadings > 0.4 are presented

**Table 2:** Factor analysis: factors and their characteristics resulting from factor analysis of items within the IPMDS

Factor	No. items in factor	Eigenvalues	Explained variance (%)	Explained variance (%; total)	Cronbach’s alpha
Basic functioning	16	14.3	31.1	31.1	0.97
Eating and digesting	6	5.2	14.1	45.2	0.76
Abnormalities at neurological examination	10	4.8	12.7	57.9	0.16

**Table 3:** Factor analysis: Spearman’s correlation coefficients between sum scores of factors

Factor	Basic functioning	Eating and digesting	Abnormalities at neurological examination
Basic functioning	1.00		
Eating and digesting	0.47*	1.00	
Abnormalities at neurological examination	0.42*	0.13	1.00

*Significant at a 0.01 level.

### Construct validity

We hypothesised that patients with a severe general MD severity would have higher median score on extracted factors compared with patients with mild disease (Table 4). This hypothesis was confirmed for factor 1 (basic functioning; *p* = 0.01; *n* = 13) but not for factors 2 (eating and digesting) and 3 (abnormalities at neurological examination (*p* = 0.07 and 0.28, respectively). In Table 5 correlation coefficients between factors, IPMDS_total_, IPMDS subdomains, severity scores, Pediatric Evaluation of Disability Index (PEDI) and the NPMDS are presented. Correlation between the basic functioning factor and PEDI, rating the functional performance and abilities of the child, was excellent (*ρ* = 0.90; *p* < 0.001); correlation between the factor abnormalities at neurological examination and rater-rated severity of abnormalities at general physical examination was weak (*ρ* = 0.23; *p* < 0.05). All predefined hypotheses used for construct validity testing indeed had good to excellent correlations (*ρ* = 0.64-0.90; Table 5).

**Table 4:** Factor analysis: median and IQR of factor sum scores for patient groups within different disease stages and difference between groups

Factor	Mild disease (*n* = 3)		Severe disease (*n* = 10)		Difference between patients mild and severe disease
	Median	IQR	Median	IQR	*P* value
Basic functioning	0	(0–0)	43	(14–56)	0.01
Eating and digesting	2	(0–2)	10	(5–16)	0.08
Abnormalities at neurological examination	3	(1–3)	10	(2–12)	0.29

*IQR* interquartile range

**Table 5:** Construct validity of the IPMDS: correlations between tests

Factor	IPMDS total	IPMDS Domain 1	IPMDS Domain 2	IPMDS Domain 3	Sum of basic functioning	Sum score of eating and digesting	Sum score of abnormalities at neurological examination
General disease severity (P)	0.806**	0.480^**^	0.558^**^	0.725^**^	0.658**	0.426*	0.038
General disease severity (R)	0.642**	0.529^**^	0.744^**^	0.810^**^	0.786**	0.476**	0.237*
Severity of abnormalities at physical examination (R)	0.853**	0.536^**^	**0.750** ^**^	0.839^**^	0.803**	0.519**	0.226*
Functional limitations (P)	0.665**	0.500^**^	0.563^**^	**0.779** ^**^	0.740**	00.328	0.135
Functional limitations (R)	0.817**	0.573^**^	0.719^**^	**0.809** ^**^	0.760**	0.381**	0.293**
PEDI total	0.912**	−0.784^**^	−0.773^**^	−**0.890** ^**^	−0.904**	−0.668**	−0.283
PEDI self-care	−0.916**	−0.767^**^	−0.767^**^	−0.881^**^	−0.882**	−0.684**	−0.263
PEDI mobility	−0.918**	−0.749^**^	−0.797^**^	−0.917^**^	−0.901**	−0.588**	−0.243
PEDI social function	−0.673**	−0.750^**^	−0.569^**^	−0.662^**^	−0.713**	−0.552**	−0.297
NPMDS total	0.840**	0.475^**^	0.771^**^	0.836^**^	0.816**	0.572**	0.183
NPMDS Section 1	0.887**	0.512^**^	0.777^**^	0.865^**^	0.882**	0.606**	0.27
NPMDS Section 2	−00.144	−0.172	−0.117	0.100	00.029	−00.133	−0.146
NPMDS Section 3	0.854**	0.597^**^	0.855^**^	0.773^**^	0.657**	0.676**	0.196
IPMDS total	1.000	0.836**	0.929**	0.907**	0.884**	0.557**	0.553**
IPMDS Domain 1	0.836**	1.000	0.551^**^	0.551^**^	0.750**	0.632**	0.439**
IPMDS Domain 2	0.929**	0.551^**^	1.000	0.883^**^	0.835**	0.615**	0.529**
IPMDS Domain 3	0.907**	0.551^**^	0.883^**^	1.000	0.931**	0.528**	0.426**

Correlation coeffients of total and subscores of the IPMDS with subjective disease severity as rated by parents and physicians (mean), with the NPMDS total and subdomain scores and with the PEDI total and subdomains scores, Bolded data represent correlations used for construct validity hypothesis testing

*PEDI* Pediatric Evaluation of Disability Index, *IPMDS* International Paediatric Mitochondrial Disease Scale,* MPMDS* Newcastle Paediatric Mitochondrial Disease Scale,* P* Parents,* R* Rater

*Significant at 0.05, **significant at 0.01 level

### Reliability

Table 6 shows the interrater reliability of the IPMDS. Inter-rater reliability of sum scores of the three factors was good (ICC_agreement_ = 0.98, 0.94 and 0.78 for basic functioning, eating and digesting, and abnormalities at neurological examination, respectively). The median ICC_agreement_ of all individual items within the IPMDS was 0.85 (0.23–0.99), 0.81 (0.44–0.98) of the first domain, 0.74 (0.23–0.93) of the second domain and 0.97 (0.93–0.99) in the third domain (Table 6).

**Table 6:** Interrater reliability of items on the International Paediatric Mitochondrial Disease Scale (IPMDS)

Factor	Basic functioning	ICC_agreement_	No. = number of patients	Feasibility
1.01	Response to environment	**0.670**	17	
1.02	Exercise capacity	**0.701**	13	x
1.03	Walking distance	**0.847**	16	
1.04	Tiredness	**0.780**	16	
1.05	Depressed	**0.847**	12	x
1.06	Epilepsy	**0.855**	17	
1.07	Headache	**0.931**	12	x
1.08	Muscle pain	0.575	12	x
1.09	Infections	0.581	17	
1.10	Chewing	**0.942**	17	
1.11	Swallowing	**0.886**	17	
1.12	Hearing	**0.762**	17	
1.13	Communicating	**0.953**	16	
1.14	Vomiting	**0.972**	17	
1.15	Gastroesophageal reflux	**0.962**	15	
1.16	Constipation	**0.833**	17	
1.17	Diarrhoea	**0.976**	16	
1.18	Cognitive functioning	0.635	15	
1.19	Behavioural problems	0.443	16	
1.20	Autistic features	0.661	11	x
1.21	Breathing subjective	**0.934**	15	
1.22a	Continence day	**0.725**	14	
1.22b	Continence night	**0.725**	14	
1.23a	Strabismus when tired	**0.736**	15	
1.23b	Ptosis when tired	0.573	15	
1.23c	Dysarthria when tired	**0.838**	13	x
2.01	Growth	**0.944**	15	
2.02	Weight	**0.751**	15	
2.03	Alertness	NV	17	
2.04	Breathing	**0.740**	17	
2.05	Dysarthria	**0.901**	12	x
2.06	Ptosis	0.553	17	
2.07	Strabismus	**0.82**	16	
2.08	Eye movements	0.691	16	
2.09	Nystagmus	**0.925**	16	
2.10	Vision	0.343	11	x
2.11	Proximal muscle power	**0.913**	15	
2.12	Distal muscle power	**0.738**	14	
2.13	Hypokinesia	0.306	14	
2.14	Abnormal movements	**0.884**	16	
2.15	Ataxia	**0.858**	14	
2.16	Tremor	**0.864**	15	
2.17	Reflexes	0.312	16	
2.18	Hypertonia	0.649	17	
2.19	Hypotonia	0.470	17	
2.20	Rigitidy	0.597	17	
2.21a	Vibration	*0.231*	11	x
2.21b	Subtle touch	NV	11	x
3.01	Communicating	**0.957**	17	
3.02	Head control	**0.983**	17	
3.03	Rolling over	**0.985**	16	
3.04	Sitting up	**0.963**	17	
3.05	Sitting up	**0.984**	17	
3.06	Standing up	**0.979**	17	
3.07	Standing	**0.974**	17	
3.08	Walking	**0.989**	17	
3.09	Running	**0.968**	13	Only >6 years
3.10	Hopping	**0.954**	13	Only >6 years
3.11	Reaching	**0.964**	16	
3.12	Grasping	**0.949**	16	
3.13	Rotating	**0.985**	14	Only >6 years

*ICC*_*agreement*_ median intraclass correlation coefficient for agreement between raters per IPMDS item: ≥ 0.7 good agreement are in bold; ≤0.3 poor agreement is in italic *x* feasible in <80 % of participants,* NV* no variance

Small pilot studies on video-rated intra-rater and test–retest reliability showed good intrarater reliability (median ICC_agreement_ = 0.87; *n* = 4). Test–retest reliability by telephone interview of the first domain after 1 week was excellent when performed by the same rater (median ICC_agreement_ = 1.0 in 15 of 16 items with variance; *n* = 4) but inconsistent when performed by a different rater (median ICC_agreement_ 0.73; *n* =4 ).

## Discussion

The IPMDS, a multidimensional scale rating clinically relevant aspects of MD in children, was developed during a Delphi-based process by an international expert team in close dialogue with patients and their parents. Designing a scale for general MD severity is challenging because of the wide variability in symptoms and (dis)abilities seen in children with MD. This complexity is reflected in the high number of items included in the IPMDS and the breath of items reflecting the multisystemic nature of the disease. Critically evaluating psychometric properties in 17 children in five international expert centres, we found good feasibility and an acceptable construct validity and reliability.

We found a suboptimal interrater reliability for some IPMDS items, mainly within the physical examination domain. This is in agreement with literature reports in which low interrater agreement in gait analysis, neurological reflexes and classification of movement disorders has been reported (Miller and Johnston 2005; Singerman and Lee 2008; Bella et al. 2012; Beghi et al. 2014). Lower agreement was observed in patients with developmental delay (Hsue et al. 2014) and in general neurologists compared with residents and experts (Beghi et al. 2014). Our teams of raters reported difficulties in agreeing on hypertonia and rigidity. This might be explained by the mixed picture of pyramidal and extrapyramidal syndromes in patients with MD (and more specifically in patients with Leigh syndrome, which represents about half of the cases included in this study). Moreover, tone is highly dependent on the child’s level of alertness, emotional state, fatigue level posture (Sanger et al. 2003), and changes in tone during the day were frequently reported by our raters.

Although the IPMDS was adopted from the NPMDS, there are important differences. First, the NPMDS consists of 26 items with mostly a 0–3 scale, whereas the IPMDS consists of 61 items, mostly on a 0–5 scale. For example, the “feeding” item in the NPMDS was replaced by items on chewing (including ability to chew, e.g. bread crusts and meat), swallowing (including difficulties/choking with dry food or fluids) and vomiting. The IPMDS therefore takes longer to execute (on average: 35 min) but is considerably more detailed. Although the burden to the patient doesn’t seem to be unacceptably high—as indicated by the positive evaluation of the IPMDS by patients and parents—the feasibility of performing the IPMDS as part of daily care at the outpatient clinic, a central tenet of developing the NPMDS, is questionable. Secondly, since behavioural problems and speech and language problems were among the most burdensome complaints experienced by patients and their parents (Koene et al. 2013b), we included these items in the complaints and symptoms and in the functional (communication) domain of the IPMDS. Thirdly, the IPMDS has a functional domain that is expected to be the most objective, responsive and relevant item for natural history and intervention studies. Lastly, we use the same scoring system for all ages (with the exception of some functional items). Although this complicates the analysis of very young children—which was also difficult in the NPMDS for 0- to 24-month-olds since newborns differ from toddlers—longitudinal analysis is more meaningful when using the same scoring system. These differences are illustrated by the lack of a statistically significant correlation between the IPMDS and the NPMDS.

Strengths of our study include: following applicable guidelines provided by the US Food and Drug administration (Administration 2009) for designing the IPMDS, the expert international team providing input to IPMDS content and validation in five expert international centres in a heterogeneous group of children with MD. However, since we aimed at a scale covering the whole phenotypic spectrum of MD, the IPMDS may contain many irrelevant items for individual patients. Disadvantages of the study include the undetermined influence of normal development on the score, and relatively small study population per centre. The small population also affects the validity of the factor analysis, so exploratory factor analysis should be repeated when more data is obtained. Lastly, eight out of 17 patients in this study had Leigh syndrome, limiting the generalizability of results.

Based on our data, we suggest using the IPMDS for assessing natural history in a larger and more heterogeneous population of children with MD. Since the IPMDS also includes subjective and functional parameters, this natural history data will not only provide clinicians with relevant information about which patients will be at risk to develop, for example, cardiomyopathy or renal failure. However, it will provide relevant prognostic information to patients and their parents. Data collected in these natural history studies should undergo another exploratory factor analysis to obtain further evidence for construct validity of the IPMDS. (We kindly invite you to upload your IPMDS scoring sheets anonymously on our website http://www.rcmm.info/ipmds). Besides, such data will facilitate setting age limits for the IPMDS, since both the number of missing items and the interrater reliability indicate that adaptations are necessary for babies and toddlers.

The aim of this study was to adapt the NPMDS to a more clinically relevant and detailed scoring system for future clinical trials in paediatric patients with MD. This is the first report on the rational for IPMDS development, including a first impression of the feasibility, reliability and validity of the current scale. Using the anonymously uploaded data from international collaborators, as well as more detailed studies on the psychometric properties in larger and more heterogeneous populations with a larger age range, we aim to optimise the scale further. Examples of future research questions include: Is the interrater reliability of Domain 2 acceptable after optimising the instructions in the manual? What is the minimal clinically important difference (Wright et al. 2012)? What is the influence of normal development and growth on the reliability and construct validity of the IPMDS, including the possible requirement of age-specific scales?

In conclusion, the IPMDS seems a robust tool for the follow-up of children with MD. Data obtained in larger and more heterogeneous populations included in natural history studies, in combination with a close dialogue with parents regarding the minimal clinically important difference, will further substantiate the instrument for clinical trials.

## Electronic supplementary material

Below is the link to the electronic supplementary material.

ESM 1 Detailed methodology of scale development and testing. (DOCX 23 kb)

ESM 2 The final version of the International Paediatric Mitochondrial Disease Scale (IPMDS). (DOC 2759 kb)

ESM 3 The final version of the manual for the International Paediatric Mitochondrial Disease Scale (IPMDS). (DOC 1393 kb)

ESM 4 (XLS 33 kb)
