# Supplementary material for: International Paediatric Mitochondrial Disease Scale
Source: J Inherit Metab Dis. 2016 Jun 9;39(5):705–12. doi: 10.1007/s10545-016-9948-7 (PMC4987390; doi:10.1007/s10545-016-9948-7)
Supplement: Supplementary file 1 — Detailed methodology of scale development and testing. (DOCX 23 kb) [file 10545_2016_9948_MOESM1_ESM.docx]

**DETAILED DEVELOPMENT AND TESTING OF THE SCALE**

*Development of the scale*

The first version of the scoring list was composed and reviewed by a team of six physicians from Nijmegen, which included three paediatricians, one paediatric resident, one paediatric rehabilitation specialist and one internal medicine physician, all seeing patients with mitochondrial diseases on a regular basis. To get a general impression of the functioning of the patient, both subjective and objective items were included. For the subjective part, we chose to interview parents and ask for their opinion on the child’s health in the past 4 weeks, similar to the NPMDS. Items in the subjective domain were based on a previous study on which sign and symptoms parents would most like to change.^1^ Besides, some items were partly adopted from existing scales, such as the NPMDS,^2^ the Newcastle Mitochondrial Disease Adult Scale (NMDAS)^3^ and the Paediatric Evaluation of Disability Inventory (PEDI).^4^ Items within the objective physical examination were dictated by the relevant general paediatric and neurological examination, including items scored with previously validated scores.^5^ Functional tests were inspired both by the prevalence and severity of disabilities within the International Classification of Functioning in Children and Youth (ICF-CY)^1^ and the domains of the Motor Function Measure (MFM).^6^ Since children under 6 years are not expected to be able to hop, this item (3.10), as well as the running item (3.9) and the rotation of a pen with a single hand (3.13), were left out of the questionnaire for children under 6 years. A manual was constructed to facilitate similar conduction of the items by all researchers.

All items were reviewed critically for completeness, relevance, uniformity, acceptability for patients and practicability by the six physicians from Nijmegen. Missing items were included. The adapted, second version of the scoring list was applied to two children with a mitochondrial disease, one child with a mild phenotype and one child with a severe condition to test for acceptability and practical difficulties. Practical adaptations were made afterwards and evaluated again by all physicians. To facilitate exclusion of items that were difficult to assess (*e.g*. headache in a severely intellectually disabled child), it was made possible to adapt the maximum score if an item was impossible to indicate.

The pilot scoring list was sent to all centres participating in this study for revision and adaptations were made on the consensus.

*Pilot study*

For the reliability and validity study of the pilot scoring list, eight patients were invited to the Radboud Center for Mitochondrial Medicine outpatient clinic. A heterogeneous group of patients was selected, with the focus on patients in which previously difficulties in performing the NPMDS were experienced. Over the course of one day, patients were scored subsequently by four physicians, with a break of 15 minutes between all consultations and a one-hour lunch break between the second and third consultation, with the aim of in keeping the programme balanced, feasible and fun for the children. The results of this scoring were used to calculate the inter-rater reliability. After the first consultation, parents were asked to fill out an evaluation form on the acceptability and the burden of the scale.

*Composition of the final scale*

Since all patients and parents evaluated the completeness and acceptability as “good”, no adaptations were made based on patient suggestions. Except for one boy with autism who was not able to complete only three out of four examinations during the pilot study because he was bored, all other children were able to complete the pilot study. Based on the experiences of the physicians involved and the inter-rater reliability, the pilot scoring list was adapted (see Supplementary Document 4 and 5). In addition, the instructions within the manual were clarified, especially for the physical examination domain. Subsequently, the list was again applied to two children with a mitochondrial disease with a mild and a severe phenotype who were not involved in the pilot study. In addition, the list was tested in patients in three other centres, who checked for acceptability and practical issues. After that, the scoring list was adapted again and sent to all participating centres. Again, adaptations were made in a Delphi-based process to compose the final scoring list, named the IPMDS (Supplementary Document 1 and 2). A 5-minute instructional video was prepared to illustrate the execution of some of the items (<https://youtu.be/WxTY-jvbFtg>).

*Testing the final scale*

Feasibility, validity and reliability studies were performed in two to four children each using the IPMDS in the Departments of Paediatrics from the Universities of Pretoria, Hong Kong, München, Rotterdam, and the Children’s Hospital of Philidelphia. Children with a mitochondrial disease, either based on pathological mutations in mtDNA or nuclear DNA or on mitochondrial dysfunction in muscle as measured by biochemistry, were eligible for inclusion. Exclusion criterion included treating physician expectation that travelling to the hospital would be too burdensome to the patient. Each individual centre selected children randomly. The number of patients and physicians was based on the local feasibility of executing the study. All raters received both written and video instructions. Each patient was seen by three or four physicians, subsequently, with appropriate pauses in between.

*Feasibility*

Feasibility was tested by asking (patients and) parents about their experiences after the first consultation and by counting the number of children in which the whole scoring list could be completed safely. The response was used as a measure for the raters’ feasibility.

*Factor analysis*

We used exploratory factor analysis to identify the underlying dimensions in the questionnaire.

*Construct validity*

To test the construct validity of the factors, the hypothesis was proposed that patients rated as having severe disease by the physicians had higher sum scores for the factors compared to patients rated as having mild disease. Since there is no gold standard for mitochondrial disease severity, we used anchors to test construct validity. The NPMDS, a measure for global mitochondrial disease severity, was performed at the second consultation in every patient. The total score of the NPMDS was correlated to the total IPMDS score. In addition, all physicians were asked to rate: i) the general severity of the mitochondrial disease; ii) the subjective severity of the mitochondrial disease as experienced by parents; iii) the abnormalities at the physical examination; iv) the functional capabilities of the child, from 0 (not severe at all) to 10 (extremely severe; visual analogue scale (VAS)) after every consultation. The PEDI was used to assess functional performance and abilities of the child. We hypothesised good correlation coefficients between: the total score of the NPMDS and the total IPMDS score; the general severity of the mitochondrial disease rated by physicians and the total IPMDS score; the subjective severity of the mitochondrial disease as experienced by parents and the IPMDS score; the abnormalities at the physical examination rated by physicians and Domain 2; and the functional capabilities of the child rated by physicians and Domain 3; the PEDI and Domain 3.

*Reliability*

Inter-rater reliability was calculated using the scores of the same patient between physicians. Intra-rater reliability was tested by re-scoring the video recording of their own interview and examinations, approximately 6 months later (2 physicians from ErasmusMC, Rotterdam). Test-retest reliability was tested by asking parents to rate the items within the first domain by telephone, both by the same rater also participating in the study (the University of Hong Kong) and between the mean score at the initial evaluation and evaluation by a nurse specialist (ErasmusMC, Rotterdam) one week later.

**REFERENCES**

1. Koene S, Wortmann SB, de Vries MC, et al. Developing outcome measures for pediatric mitochondrial disorders: which complaints and limitations are most burdensome to patients and their parents? Mitochondrion 2013;13:15-24.

2. Phoenix C, Schaefer AM, Elson JL, et al. A scale to monitor progression and treatment of mitochondrial disease in children. Neuromuscul Disord 2006;16:814-820.

3. Schaefer AM, Phoenix C, Elson JL, McFarland R, Chinnery PF, Turnbull DM. Mitochondrial disease in adults: a scale to monitor progression and treatment. Neurology 2006;66:1932-1934.

4. Feldman AB, Haley SM, Coryell J. Concurrent and construct validity of the Pediatric Evaluation of Disability Inventory. Phys Ther 1990;70:602-610.

5. Van Dillen LR, Roach KE. Interrater reliability of a clinical scale of rigidity. Phys Ther 1988;68:1679-1681.

6. Berard C, Payan C, Hodgkinson I, Fermanian J. A motor function measure for neuromuscular diseases. Construction and validation study. Neuromuscul Disord 2005;15:463-470.
