# Supplementary material for: International Paediatric Mitochondrial Disease Scale
Source: J Inherit Metab Dis. 2016 Jun 9;39(5):705–12. doi: 10.1007/s10545-016-9948-7 (PMC4987390; doi:10.1007/s10545-016-9948-7)
Supplement: Supplementary file 2 — The final version of the International Paediatric Mitochondrial Disease Scale (IPMDS). (DOC 2759 kb) [file 10545_2016_9948_MOESM2_ESM.doc]

**
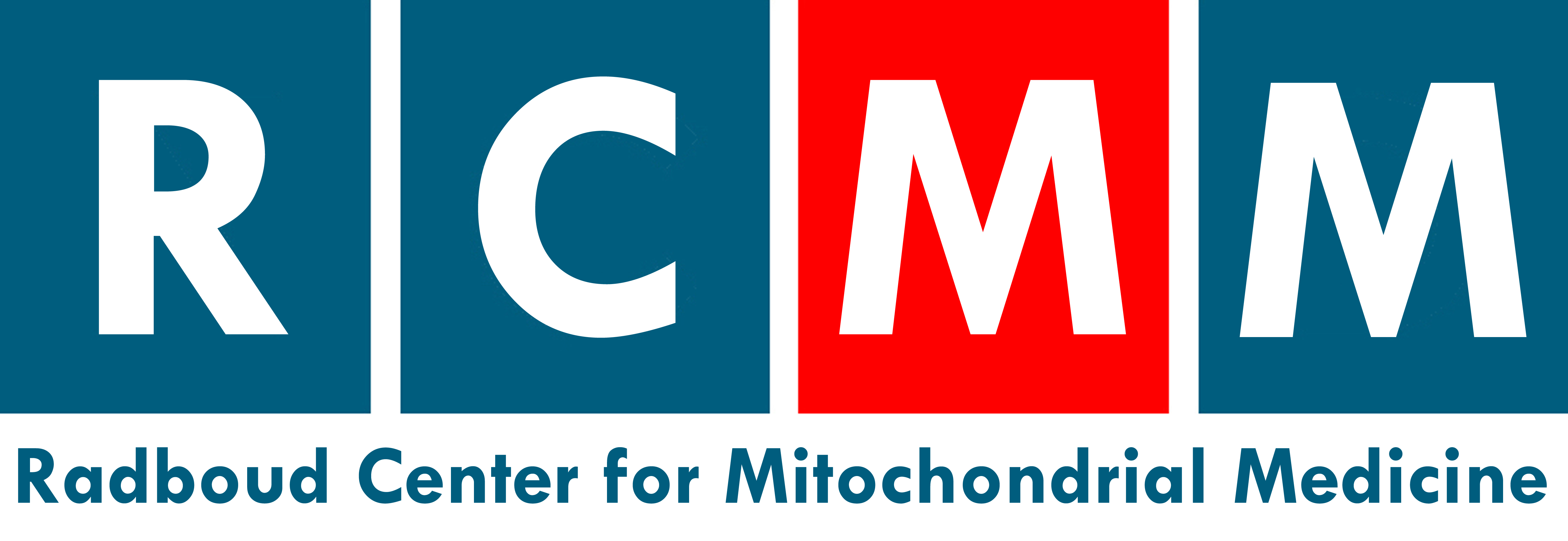
**

**International Paediatric Mitochondrial Disease Scale (IPMDS)**

**Name:**

**Date of birth:**

**Date of assessment:**

**Time since previous IPMDS:**

**Name physician:**

**Domain 1: (raw score) /(103 - ) = %**

**Domain 2: (raw score) /(76 - ) = %**

**Domain 3: (raw score) /(64 - ) = %**

**Total score: (raw score) /(243 - ) = %**

**
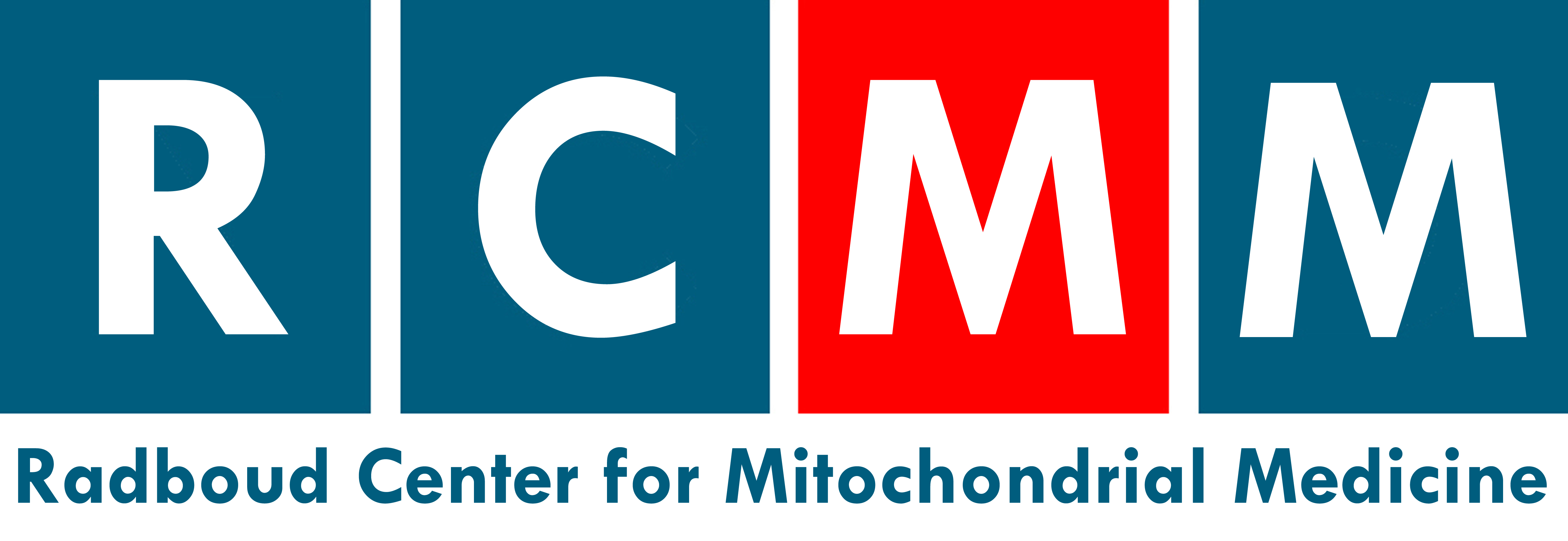
**

**International Paediatric Mitochondrial Disease Scale (IPMDS)**

**Anonymous**

**ID (for follow-up purposes):**

**Age:**

**Gender:**

**Ethnicity:**

**Clinical diagnosis:**

**Biochemical diagnosis:**

**Genetic diagnosis:**

**Date:**

**Name physician:**

**Disease course since previous IPMDS**

**Parents: Stable Improving Deteriorating**

**Physician: Stable Improving Deteriorating**

**We kindly ask you to upload your PDF at** [**http://www.rcmm.**](http://www.rcmm./)**info/ipmds to facilitate data collection**

**Over the past 2 days:**

**1. How is your child’s physical condition?**

A Stable

B Deteriorating

C Improving

**2. How is your child’s mental health?**

A Stable

B Deteriorating

C Improving

**3. In general, how happy is your child feeling?**

A Unhappy

B Not happy nor unhappy

C Happy

D Very happy, more happy than others

**4. In general, how comfortable is your child?**

A Uncomfortable

B Not comfortable nor uncomfortable

C Comfortable

**1. Complaints and symptoms**

**Please answer the following questions with regard to how your child was complaining over the past 4 weeks:**

**1. Which of the following best describes your child’s alertness and response to his/her environment?**

0 Appropriately alert during daytime hours; good response to the environment

1 Alternates between periods of sleepiness and full alertness and/or requires stimulation to remain alert

2 Sleepy, only reacting to touch, noise or visual stimulation

3 Alternating between periods with no response to environment and periods of sleeping

**2. How well has your child been able to perform exercise?**

0 Normal compared to peers

1 Limited in playing sports, participating in (adapted) physical education

2 Symptomatic on inclines or stairs

3 Adaptations to save energy required for playing outside

4 Adaptations to save energy required for playing inside

5 Restricted on playing inside and frequent resting required despite adaptation of playing inside

* Not playing due to severe cognitive impairment

**3. For how long is your child able to walk?**

0 Normal compared to peers

1 Rest required or impaired coordination after 1 hour of walking

2 Rest required or impaired coordination after 30 minutes of walking

3 Rest required or impaired coordination after 15 minutes of walking

4 Rest required or impaired coordination after 5 minutes of walking

5 Not at all

**4. How tired has your child been?**

0 Normal

1 Tired *the day after* going on an exciting day out e.g. an amusement park or zoo (with or without use of wheelchair)

2 Not able to go on an exciting day out e.g. a amusement park or zoo (with or without use of wheelchair) because of tiredness

3 Not able to go on half day family trips e.g. to park or playground (with or without use of wheelchair) because of tiredness

4 Not able to go to school for a full week because of tiredness

5 Not able to go to school at all because of tiredness

**5. Has your child been depressed, sad, withdrawn, anxious or quiet?**

0 Not more than peers

1 Only in specific circumstances (tired, exciting events, setbacks, life events)

2 Symptoms present every week

3 Symptoms present every day but not limiting school performance or the ability to make friends

4 Symptoms limiting school performance or the ability to make friends

5 Severe depressive symptoms requiring hospitalization

* Impossible to indicate due to severe cognitive impairment

**6. Has your child experienced epileptic seizures? At the moment these are treated / untreated * (*please circle)**

0 No

1 Once with self recovery this month (seizure lasted sec/min)

2 Two or three times with self recovery this month, or absence epilepsy

3 One convulsion in which emergency medication was necessary or where there was no recovery within 5 minutes, or more than 3 with self recovery last month

4 Multiple or prolonged convulsions in which emergency medication was given or where there was no recovery within 5 minutes

5 Hospitalization or more than 3 generalized convulsions (not absences) a week

**7. Has your child suffered from headache?**

0 No

1 Mild headaches not limiting daily activities

2 Daily activities limited by headaches less than once a week

3 Daily activities limited at least once a week or true migraine attacks at least once a month

4 True migraine attacks despite chronic treatment or true migraine attacks at least once a week

5 Chronic migraine: true migraine headaches 15 days/month for longer than 3 months

* impossible to indicate

**8. Has your child suffered from muscle pain?**

0 No

1 Occasional muscle pain after exercise not limiting daily activities

2 Recurrent mild muscle pain after exercise not limiting daily activities

3 Muscle pain after exercise limiting daily activities

4 Spontaneous muscle pain (not only after exercise) limiting daily activities

5 Severe, spontaneous muscle pain

* impossible to indicate

**9. Has your child suffered any infections?**

0 No or only mild infection

1 Infection with subsequent tiredness limiting daily activities for more than one day

2 Infection with subsequent tiredness limiting daily activities for more than one week

3 Infection taking more than one week, with subsequent school absence for more than one day

4 Infection taking more than one week, with subsequent school absence for more than one week

**10. Has your child experienced problems with chewing?**

0 No

1 Bread-crusts and meat are tiring

2 Bread-crusts and meat are avoided because of the chewing problems

3 Food is pureed because of the chewing problems

4 Pureed food is supplemented with tube feeding because of chewing difficulties

5 Only tube feeding because of chewing problems

**11. Has your child experienced problems with swallowing?**

0 No

1 Difficulties (choking) with fluids or dry food

2 Difficulties (choking) with all foods; adaptation of the diet

3 Choking despite adaptation of the diet (e.g requiring supplementary tube feeding)

4 Solely tube feeding because of choking

5 Spontaneous choking on saliva, or recurrent aspiration pneumonia

**12. Has your child experienced hearing problems?**

0 No

1 Proven hearing loss, without the need for a hearing aid or fully corrected with hearing aid

2 Not fully corrected with hearing aid, but no impaired communication

3 Not fully corrected with hearing aid, impaired communication

4 Reaction to loud sounds (e.g. clapping) only, despite use of a hearing aid

5 No reaction to loud sound

**13. Has your child found problems in communicating?**

0 Normal, age appropriate communication

1 Stammer, dysarthria or language delay impairing communication with strangers

2 Stammer, dysarthria or language delay impairing communication with parents

3 Effective communication only using alternative methods (speech computer, sign language)

4 No effective communication with strangers despite using appropriate alternative methods

5 No effective communication with parents despite alternative methods

**14. Has your child been vomiting?**

0 No frequent vomiting

1 Vomiting at least once a week

2 Vomiting once a day despite adaptation of feeding

3 Vomiting more than once a day despite adaptation of feeding

4 Continuous tube feeding

5 Malnourished as a consequence of vomiting despite adaptations in feeding

**15. Has your child been having symptoms of (or medication for) gastroesophagal reflux?**

0 No symptoms of gastroesophageal reflux (heartburn, regurgitation, uncomfortable when lying down after consumption of food)

1 No symptoms with one medication

2 No symptoms with multiple medications

3 Symptoms despite multiple medications

4 Esophageal erosions despite treatment

5 Fundoplication or malnourished despite adaption of feeding

* impossible to indicate

**16. Has your child had symptoms of constipation?**

0 No constipation

1 Variable defecation pattern with defecation between 2x/week and 3x/day

2 Constipation fully resolved with oral medication

3 Constipation not fully resolved with oral medication (frequency 1-2x per week)

4 Constipation with frequency <1x/week despite oral medication

5 Constipation requiring clysters or colonic lavage

**17. Has your child had symptoms of diarrhoea?**

0 No diarrhoea

1 Loose stool ≥ 3x/day

2 Shapeless or watery stool < 3x/day

3 Shapeless or watery stool ≥ 3x/day

4 Episodes of diarrhoea that require a change of clothes, occurring at least once per week

5 Severe diarrhoea necessitating increased fluid supply

**18. Cognitive development**

0 Normal cognitive development, mainstream school or attending special school only because of physical disability

1 Attending special school because of mental disability but learning new skills

2 Attending special school because of mental disability but *not* learning new skills

3 Losing skills in one area (cognitive, language, motor, social/emotional)

4 Losing skills in more than one area or severe cognitive impairment due to prior loss of skills

**19. Does your child have behavioural problems?**

0 Normal compared to peers

1 More severe behavioural problems compared to peers

2 Behavioural problems limiting the ability of the family to go out

3 Behavioural problems limiting the ability to make friends

4 Behavioural problems limiting the ability to go to school

5 Not attending school because of behavioural problems

**20. Does your child have autistic features?**

0 Normal compared to peers

1 No official diagnosis of an autism spectrum disorder. Parents do recognize autistic features which cannot be explained by mental retardation, in their child

2 Official diagnosis of an autism spectrum disorder. Limited social interaction, limited flexibility of behavior or difficulty switching between activities requiring support, but not limiting daily functioning

3 Official diagnosis of an autism spectrum disorder. Clear problems in social interaction, difficulty with change and limited flexibility in behavior, limiting daily functioning

4 Official diagnosis of an autism spectrum disorder. Severe deficit in social communication, great difficulty with change and very limited flexibility in behavior despite the use of medication

* impossible to indicate due to severe cognitive impairment

**21. Breathing pattern**

0 Normal breathing pattern

1 Frequent sighing or irregular breathing

2 Nocturnal ventilator support required

3 Periods of apnoea (> 20 sec) or continuous ventilation

**22. Has your child been continent of urine (able to indicate when toilet visit is needed > 90% of time)?**

During the day Yes (0) /No (1)

During the night Yes (0) /No (1)

**23. Does your child experience any of the following items when tired?**

Strabismus (squint) Yes (1) /No (0)

Ptosis (drooping eyelids) Yes (1) /No (0)

Dysarthria (impaired speech) Yes (1) /No (0)

**2. Physical examination**

*In the case of asymmetrical abnormalities, score the most severely affected side*

**1. Growth**

0 Normal (-2 to +2 SD) for target height/catch up growth

1 Growing parallel to growth curve, but below -2SD for target height

2 Deviating from his/her own growth curve appropriate for target height

3 Deviating from his/her own growth curve, and below -2SD for target height

**2. Weight for height**

0 Normal or catch up growth when under -2 SD

1 Growing parallel to -2 SD or deviating (≥ 1 SD) in positive direction in children above +2 SD

2 Deflecting (≥ 1 SD) from growth curve in negative direction

3 Unintentionally losing ≥3% weight compared to the last measurement

**3. Alertness**

0 Eyes opened spontaneously

1 Opens eyes when talking (verbal child) or to parent’s voice

3 Opens eyes when touched

3 Opens eyes on pain stimulus

4 Not opening eyes

**4. Breathing pattern**

0 Normal breathing pattern

1 Sighing or irregular breathing observed during physical examination

2 Periods of apnoea (> 20 sec) during examination

**5. Dysarthria**

0 No dysarthria

1 Nasal, hoarse, low pitch voice, or irregular speech, but easily understood

2 Obvious dysarthria, understood more than 50% of the time

3 Obvious dysarthria, understood less than 50% of the time

4 Not able to communicate verbally or use of alternative methods ***primarily*** due to dysarthria

* Not able to communicate due to other causes

**6. Ptosis**

0 Normal

1 Mild ptosis not obscuring either pupil

2 Unilateral ptosis obscuring > 1/3 of pupil

3 Bilateral ptosis obscuring > 1/3 of pupils

4 Bilateral ptosis obscuring > 2/3 of pupils, or previous ptosis surgery

**7. Strabismus**

0 Normal

1 Permanent strabismus of one eye

2 Permanent strabismus of both eyes

**8. Eye movements**

0 Normal

1 Some restriction in any of the eye movements with normal abduction

2 Partial abduction possible

3 Abduction minimal

**9. Nystagmus**

0 No or physiological (endpoint) nystagmus

1 Gaze evoked nystagmus

2 Spontaneous nystagmus

**10. Vision – using glasses and both eyes**

0 ≥ 1.0 (Snellen or crowded LogMAR chart) or attention to small objects at a distance

1 ≥0.5 – <1.0 (Snellen or crowded LogMAR chart)

2 ≥0.1- <0.5 (Snellen or crowded LogMAR chart) 3 < 0.1 (Snellen or crowded LogMAR chart)

4 Only reaction to big, colored objects within reach

5 No reaction to big, coloured objects within visual field

* Impossible to indicate due to severe cognitive impairment

**11. Proximal muscle strength**

0 Normal power in arms and legs

1 Moves legs and arms against resistance, but not against full resistance

2 Moves arms and legs against gravity, but not against resistance

3 Active joint movement when gravity is eliminated (e.g. in horizontal plane)

4 Contraction of muscle is visible, but no movement of the joint

5 No contraction visible

**12. Distal muscle strength**

0 Normal power in feet and hands

1 Moves hands and feet against resistance, but not against full resistance

2 Moves hands and feet against gravity, but not against resistance

3 Active joint movement when gravity is eliminated (e.g. on a underground)

4 Contraction of muscle is visible, but no movement of the joint

5 No contraction visible

**13. Hypokinesia**

0 Absent or reduced facial expression due to other causes (e.g. facies myopathica)

1 Reduced facial expression only, no slowing of body movements

2 Reduced facial expression, mild slowing of body movements

3 Significant slowing of body movements

**14. Abnormal, involuntary movements and abnormal posturing**

0 Absent

1 Intermittent involuntary movements of one extremity, not interfering with daily activities

2 Intermittent involuntary movements of multiple extremities, mildly interfering with daily activities

3 Continuous involuntary movements of whole body, markedly interfering with daily activities

**15. Ataxia**

0 Absent

1 Impaired coordination (dysmetria) compared to peers on examination only (hesitant heel-toe, disturbed alternate movements)

2 Gait reasonably steady, but unable to maintain heel-toe, mild dysmetria, truncal ataxia, not limiting the ability to sit

3 Ataxic gait, not able to walk 2 passes heel-toe, past pointing with intention tremor, truncal ataxia, limiting the ability to sit

4 Walking unsafe or sitting impossible without support, ***primarily*** due to ataxia

**16. Tremor**

0 Absent or physiological tremor

1 Tremor present at rest, but not affecting coordination

2 Tremor affecting coordination

3 Unable to grab things ***primarily*** due to tremor

**17. Reflexes**

0 Normal, brisk, indifferent or hypoactive reflexes

1 Abnormally brisk reflexes

2 Expansion of the reflex zone or crossed adductor response

3 Non sustained clonus

4 Sustained clonus

**18. Hypertonia**

0 Normal tone or hypotonic

1 Increased tone, catch followed by relaxation or minimal resistance at the end of the range of motion

2 Increased tone, catch followed by minimal resistance during less than the half of the range of motion

3 Resistance during most of the whole range of motion

4 Resistance, passive movements are difficult

5 Rigid flexion or extension

**19. Hypotonia**

0 Normal tone or hypertonic

1 Mild slipping through/head lag or hypotonia or hypermobility on physical examination

2 Obvious slipping through/head lag, sits with curved back

3 Obvious slipping through/head lag, difficulty maintaining the head in midline position while sitting with lower backsupport

4 Obvious slipping through/head lag, unable to sit with or without backsupport only ***primarily*** due to hypotonia

**20. Rigidity**

0 No rigidity or hypertonia only

1 Paratonia

2 Mild and inconsistent resistance throughout the range of motion

3 Resistance is detected consistently during the range of motion, but full range of motion is obtained easily

4 Resistance to obtain passive movement requires maximal effort by the rater or full range of motion is not obtained ***primarily*** due to rigidity

**21. Sensory examination**

Vibration Normal (0) / Abnormal (1) / Absent (2)/Impossible to indicate (*)

Subtle touch Normal (0) / Abnormal (1) / Absent (2) /Impossible to indicate (*)

**3. Functional tests**

*Some, indicated items are only for children ≥ 6years*

**1. Communication**

0 Actively interacting with researcher, easily understood, follows complex instructions

1 Actively interacting with researcher; difficulty expressing self; uses alternative methods to communicate; follows simple instructions

2 Actively interacting with researcher; difficult to understand despite use of alternative communication methods; child has difficulty understanding simple instructions

3 Reactive to researcher but no understanding of simple single-level instruction and not understood by researcher

4 Reacts only to tactile, auditory or visual stimulation

5 No reaction to tactile, auditory or visual stimulation

**2. Head control – maintains head in midline while sitting with back support**

0 Normal

1 Control, but only with back support

2 Control longer than 1 min

3 Control between 30 seconds and 1 min

4 Control shorter than 30 seconds

5 Absent

**3. Rolling over – from supine to prone and back**

0 Able to roll over from supine to prone and back over both sides

1 Able to roll over from supine to prone and back, but only to left or right

2 Able to roll over from supine to prone and back, but only to supine or prone

3 Initiates movement and lifts shoulder and hip from underground

4 Initiates movement and moves arm over the midline

5 No attempt to roll over

**4. Sitting up – from lying supine to sitting**

0 Able to sit up without help of elbows or hands

1 Able to sit up with help of elbows or hands

2 Able to sit up using a trick manoeuvre (e.g. turns to prone side)

3 Lifts head from ground, but unable to sit up

4 Not able to lift head from the ground

5 No attempt to sit up

**5. Sitting position – sits without back support**

0 Normal

1 Able to sit without support for at least 5 seconds, but not able to hold arms upright for 5 seconds

2 Able to sit without support, but for less than 5 seconds

3 Able to sit only with support of the hands

4 Not able to sit

**6. Standing up from a chair [chair at a height that allows both feet to be resting flat on the ground] if possible without support of hands**

0 Normal

1 Compensatory movements, but within normal time and no help of hands

2 Slips off, needs own hands for support or abnormal method, but within normal time

3 Takes longer than expected for age, due to cognitive or motor inabilities

4 Only possible with help of caretaker, or unsafe procedure

5 Not able to stand up

**7. Standing** **– stands without support**

0 Normal

1 Compensatory movements or abnormal posture

2 Stands for less than one minute without support

3 Stands for less than 30 seconds without support

4 Stands (>10 seconds) only with support

5 No attempt

**8. Walking – walking without support**

0 Normal

1 Compensatory movements or abnormal posture

2 Walks for more than 10 m, but not for 20 m

3 Walks for less than 10 m

4 Walks only a few (1-3 steps) or only walks with support

5 No attempt to walk or not able to walk with support

**9. Only ≥ 6years: Running**

0 Normal

1 Normal, but slower

2 Compensatory movements or abnormal posture

3 Decreased time of lifting both feet from the floor (levitation)

4 Increases speed but no levitation (moment that both feet are lifted from the floor)

5 Absent

**10. Only ≥ 6years: Hopping on one foot – 10 times**

0 Normal

1 Normal but decreasing hopping height during exercise; OR able to hop normally more than 5 times

2 Hops 10 times, starts with toes from the floor, finishes not lifting toes from the floor; OR able to hop normally more than 3 times

3 Hops 10 times but not lifting toes from the floor

4 Stands on one foot

5 No attempt

**11. Reaching – grasp pen/small toy held in the air by the examiner within arm length of the patient**

0 Normal

1 Abnormal movements

2 Abnormal movements, much slower to reach object

3 Possible to approach the item, but not grab it

4 Only inefficient/uncoordinated efforts not approaching the item

5 No attempt

**12. Grasping – grasps pen within arm length from the table and transfers pen/small toy to other hand**

0 Normal

1 Abnormal movements, but able to achieve both grasping and transferring

2 Able to grasp but not transfer

3 Able to approach the item, but not grasp it

4 Only inefficient/uncoordinated efforts not approaching the item

5 No attempt

**13. Only ≥ 6 years: rotates pen within the hand with fingers**

0 Normal

1 Rotates the pen, but with compensatory movements or tricks

2 Initiates rotating movement, but unable to turn pen around

3 Opens hand with pen in it, but unable to initiate rotating movement

4 Attempts, but fails to open hand

5 No attempt
