# Supplementary material for: International Paediatric Mitochondrial Disease Scale
Source: J Inherit Metab Dis. 2016 Jun 9;39(5):705–12. doi: 10.1007/s10545-016-9948-7 (PMC4987390; doi:10.1007/s10545-016-9948-7)
Supplement: Supplementary file 3 — The final version of the manual for the International Paediatric Mitochondrial Disease Scale (IPMDS). (DOC 1393 kb) [file 10545_2016_9948_MOESM3_ESM.doc]

**
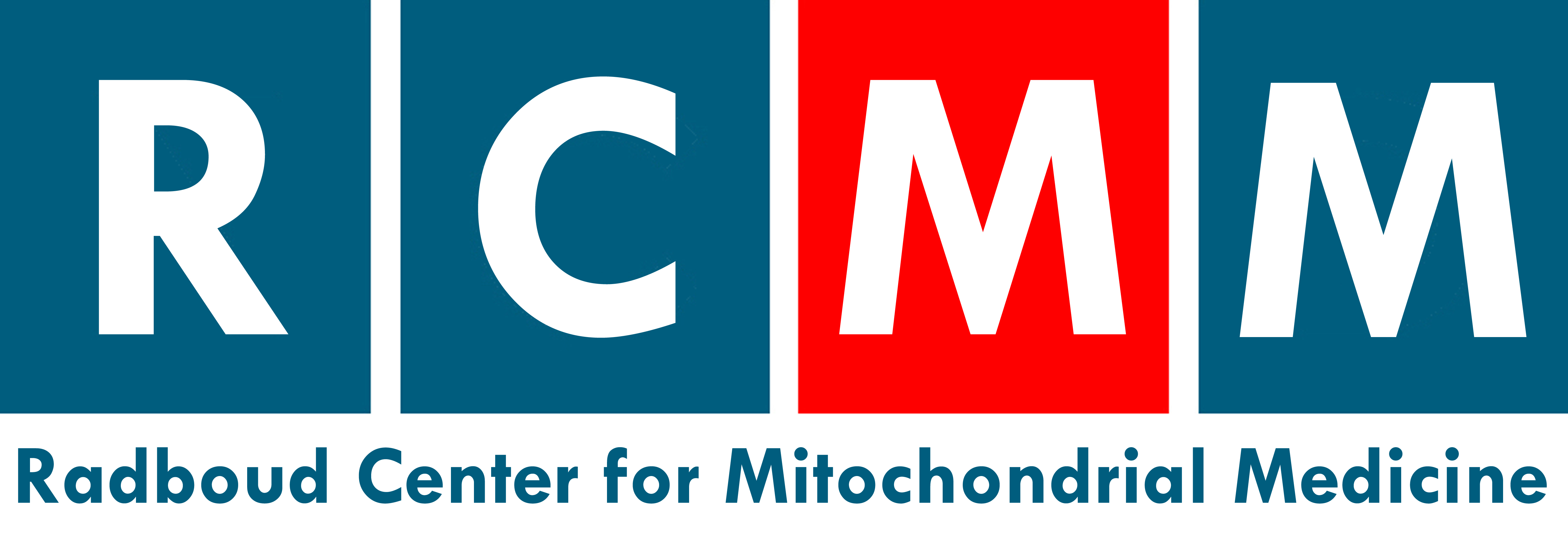
**

**International Paediatric Mitochondrial Disease Score (IPMDS)**

**MANUAL**

**MANUAL**

**Introduction**

The IPMDS was designed to monitor general disease progression of symptoms associated with mitochondrial disease in children (aged 0 – 18 years) with a mitochondrial disorder. The scale provides detailed insight into the complaints and symptoms (domain 1), physical examination (domain 2) and functioning of the patient (domain 3).

**PREREQUISITS**

**TRAINING**

In order to familiarize examiners with the utilization of the IPMDS and its scoring principles, a training session, in which the items are practiced in a in a role-play at least twice, is recommended.

Please also watch our instructional video.

**MATERIAL**

- A complete growth chart of the child

- A Snellen or crowded LogMAR chart at least 3 m from a wall

- A physiotherapy mat or a wide examination table

- A chair (with adjustable height if possible). The height of the seat should be such that the person’s feet touch the floor or footstool when seated (with or without footstool)

- A footstool if indicated

- A pillow for back support if indicated

- Space within a corridor, wide at least 2m, to enable free movement of 20 m, with marks at 10 and 20 m

- A marked line traced on the floor, approximately 3 m long and 2 cm wide.

- A penlight

- A reflex hammer

- A tuning fork

- A pen

**PATIENT**

The patient should be wearing comfortable clothes, which will not interfere with movements. Corsets should be removed, if possible. Patients should be barefooted without orthoses and socks.

**SPECIFIC INSTRUCTIONS**

The order of the items should be respected.

**DOMAIN 1**

The questions in domain 1 should be assessed with a caregiver/patient interview and ***not*** by chart review **nor** the physician’s own judgment.

All questions enquire about the severity of the complaints and symptoms over the **past 4 weeks**.

The answer “normal” should always be verified by asking for the symptoms rated 1 or 2 points (e.g in question 1 “So he doesn’t need stimulation to remain alert? And there are no periods of sleepiness during the day?”).

**DOMAIN 2**

The items in domain 2 are assessed by physical examination. In the case of asymmetrical abnormalities, the worse side is scored. If the item is difficult to interpret, please note this on the scoring sheet.

Item specific instructions

*Item 1: Growth*

The height curve of the past 6 months is used to determine this item.

The individual target height of the child is calculated as following

- - Girls: (height father + height mother – 13) / 2 + 4.5 ( in cm)
  - Boys: (height father + height mother + 13) / 2 + 4.5 ( in cm)

*Item 2: Weight for height*

The weight for height curve of the past 6 months is used to determine this item.

*Item 3: Alertness*

No specific instructions, may be observed during the completion of domain 1

*Item 4: Breathing pattern*

No specific instructions, sighing and breathing pattern may be observed during the completion of domain 1

*Item 5: Dysarthria*

May be observed during the completion of domain 1. The type of dysarthria (spastic, myopathic, extrapyramidal, ataxic) is not relevant, the consequences for communication are. If the child is not able to communicate for other reasons, score a *.

*Item 6: Ptosis*

Ptosis is observed when the child is looking forward with his/her head in a neutral position. Ask for previous ptosis surgery.

*Item 7: Strabismus*

The light reflex in both eyes is observed when looking forward. In case of strabismus, the light reflex will be assymetrical (not on the pupil).

*Item 8: Eye movement*

The child is asked or stimulated to follow a penlight or an interesting object from left to right; up and down; and up and down when the eyes are deviated left and right. Especially the abduction (pull out) of both eyes is observed.

*Item 9: Nystagmus*

Nystagmus is observed by looking at the blood vessels on the sclera or the iris during the eye movement examination. Physiological nystagmus (small-amplitude (<2°) conjugate jerk nystagmus on far eccentric gaze (>40°)) is rated as a 0. When nystagmus is observed when the child’s looking forward, this is noted as spontaneous nystagmus.

*Item 10: Vision*

The child sits or stands at a known distance (e.g. 3m) from the Snellen chart with both eyes opened and using usual glasses. Visual acuity is calculated as (distance to chart (e.g. 3m) / number behind last row adequately seen). If the child is not able to read, the circle (O) can be searched for (“Is this a circle?”) or the response to visual stimuli is noted. In case of cognitive impairment, note the reaction to “interesting objects” at a distance.

*Item 11: Proximal muscle strength*

The m. biceps brachii and the m. quadriceps femoris are tested bilaterally. The strength of the child is compared to peers when differentiating between a score of 0 and 1. Gravity is eliminated by asking to move the joint in a horizontal plane on a surface.

*Item 12: Distal muscle strength*

The grip strength and the m. tibialis anterior are tested bilaterally. The strength of the child is compared to peers when differentiating between a score of 0 and 1. Gravity is eliminated by asking to move the joint in a horizontal plane on a surface.

*Item 13: Hypokinesia*

The facial expressions are observed during interaction. Decreased facial expression due to other causes (e.g. facies myopathica) is not rated in this item. Slowing of body movements (hypokinesia) is observed during the other items of the physical examination, walking or playing.

*Item 14: Abnormal, involuntary movements and abnormal posturing*

The presence of dystonia (abnormal positioning of e.g. hands), chorea (quick, jerky movements), athetosis (slow, writhing movements) or hemiballismus (unilateral, flinging movements) is assessed. The effect of these movement disorders on coordination is observed during (un)dressing, walking or playing.

Tics and tremor are not scored at this item.

*Item 15: Ataxia*

Ataxia is tested by heel-toe, alternate movements, the tap nose test and – if abnormal - the ability to sit. The performance on the heel-toe and alternate movements should be compared to peers. Abnormal coordination without ataxia is not rated in this item.

*Item 16: Tremor*

The presence of a tremor is tested by asking the child to put both hands (palm down) forward with straight arms. A physiological tremor is defined as a tremor of 10 Hz.

*Item 17: Reflexes*

Deep tendon reflexes (patellar reflexes, ankle jerk reflexes and biceps brachii reflexes) including reflex spread are tested bilaterally. Physiologically brisk but normal reflexes are rates as a 0. Expansion of the reflex zone is reflex contraction after tapping on the tibia instead of the knee tendon. The crossed conductor response is reflex contraction of the right muscle when tapping on the left knee tendon (and vice versa). The worse side is scored.

*Item 18: Hypertonia*

The presence of hypertonia is assessed by flexion and full extension of the ankles, knees and elbows bilaterally, at slow and at fast speed when the child is relaxed or distracted. The presence of spastic resistance or a catch is observed. Contractures are looked for by fully extending and flexing the ankles, knees and elbows.

*Item 19: Hypotonia*

Muscle tone is assessed by holding the child under the axillae and by placing the child in sitting position. In older children, the tone is assessed during passive movement of the elbow and observing the sitting position.

*Item 20: Rigidity*

The presence of hypertonia is assessed by flexion and full extension of the ankles, knees and elbows bilaterally, at slow speed when the child is relaxed or distracted. The presence of (cogwheel) rigidity is rated. Paratonia, the active resistance of the patient to any movement despite distraction, often augmented by increasing the speed of the movement and often interpreted as “poor cooperation”, is rated as a 1. When the full range of motion cannot be obtained due to contractures or spasticity, the rigidity over the remaining free trajectory is rated.

*Item 21: Sensory examination*

Vibration and subtle touch are tested on the big toe of both feet. The child is asked to close his eyes and say if he can feel that he’s being touched, in children not able to respond appropriately, the reaction to the stimulus is noted.

**DOMAIN 3**

In domain 3, the functional abilities of the child are assessed. For this domain, the reason why the child is not able to sit (PMR, ataxia, hypotonia, contractures) is not relevant.

Three attempts are allowed if the investigator expects the child to be able to perform better. Using tricks during three attempts is rated as “compensatory movements”. If the child refuses to attempt the item, if the item was forgotten, if the item is not safe to execute or if the child is not able to maintain the starting position, the score for the item is 5. “Refusal” or “forgotten” should be noted on the scoring sheet.

Some items are dependent on the normal development of the child and are therefore only applicable to patients aged 6 years and older. This is indicated at the item (item 10 an 13).

Investigators are allowed to give an example of the intended movement at every attempt. The instructor is allowed to show the movement by moving the child’s limb in the intended direction (e.g. by letting the pen rotate in his/her hand and asking the child to continue).

Item specific instructions

*Item 1. Communication*

As observed during the assessment of domain 1 and 2. Complex instructions are double instructions (do this followed by that) such as the verbal instructions for the dysdiadochokinesis and ataxia exercises. Simple instructions include single instructions (get on the examination table). The cause of the difficulty expressing him/herself (PMR, autism, dysarthria) is not relevant.

*Item 2. Head control*

Adequate back support is a straight back of a (wheel)chair, supporting at least ¾ of the back, but not more than shoulder height. The researcher is allowed to position the head above the pelvis, if necessary; the ability to remain the head in this position is scored.

*Item 3. Rolling over*

Child is lying flat on his/her back with the head in the midline on a broad examination table or on a hard mat on the ground. The child is asked or tempted (with an interesting object) to roll over to both sides and back.

*Item 4. Sitting up*

Child is lying flat on his/her back on a hard mat on the ground. The child is asked or encouraged (with an interesting object) to sit up, if possible without hands (e.g. by asking the child to stab the arms forward).

*Item 5. Sitting position*

The child sits without backsupport on a hard mat on the ground. The child is asked to sit without support for 5 seconds and then hold their arms upright for 5 seconds.

*Item 6. Standing up from a chair*

The child sits at a chair with a hard surface at a height that allows both feet to be resting flat on the ground or on a footstool. The hands are resting on the lap of the child. The child is asked to raise from the chair without using the hand (e.g. by placing them on their head).

*Item 7. Standing*

The child stands on a hard surface in a room or corridor of at least 2m wide. The researcher is behind of the child to catch him/her if he falls, but so that the child cannot seek physical support.

The child is asked to stand straight for one minute.

*Item 8. Walking*

The child stands on a hard surface in a room or corridor of at least 2m wide. The researcher is behind of the child to catch him if he falls but so that the child cannot seek physical support.

The child is asked to walk for 20 m at his/her usual pace.

*Item 9. Only ≥ 6years: Running*

The child stands on a hard surface in a room or corridor of at least 2m wide. The researcher is behind of the child to catch him if he falls but so that the child cannot seek physical support.

The child is asked to run or walk as fast as possible for 20 m.

*Item 10. Only ≥ 6years: Hopping on one foot*

The child stands on a hard surface in a room or corridor of at least 2m wide. The researcher is behind of the child to catch him if he falls but so that the child cannot seek physical support.

The child is asked to hop on one leg for 10 times. One hop is take off and landing at the same single foot.

*Item 11. Reaching*

The child sits stably in a (wheel)chair with adequate back support at a height that allows both feet to be resting flat on the ground or on a footstool. The table is at the height of the elbow or maximally halfway the height of the elbow and the shoulder in resting position. The child is asked or encouraged to reach for a pen or a toy of approximately similar size and weight. The pen/toy is held in the air within arms length of the child.

*Item 12. Grasping*

The child sits stably in a (wheel)chair with adequate back support at a height that allows both feet to be resting flat on the ground or on a footstool. The table is at the height of the elbow or maximally halfway the height of the elbow and the shoulder in resting position. The child is asked or encouraged to grasp a pen or a toy of approximately similar size and weight from the table and to put it in his/her other hand and back. The pen is placed within arm length of the child.

*Item 13. Only ≥ 6 years: Rotating*

The child sits stably in a (wheel)chair with adequate back support at a height that allows both feet to be resting flat on the ground or on a footstool. The table is at the height of the elbow or maximally halfway the height of the elbow and the shoulder in resting position.

Rotating the pen is turning the pen around in the hand like a majorette (see instructional video).

**INTERPRETATION**

The score is expressed as the percentage of items which were feasible to perform. Asterixes (*) can be scored as well, the total score will change accordingly. E.g. if the parents are not able to indicate the presence of headache, the maximum score of the first domain changes from 103 to 73. If the child is not cooperative during the execution of domain 2 and 3, these items are omitted from the total score.
